# Determinants of childhood immunisation coverage in urban poor settlements of Delhi, India: a cross-sectional study

**DOI:** 10.1136/bmjopen-2016-013015

**Published:** 2016-08-26

**Authors:** Niveditha Devasenapathy, Suparna Ghosh Jerath, Saket Sharma, Elizabeth Allen, Anuraj H Shankar, Sanjay Zodpey

**Affiliations:** 1 Indian Institute of Public Health, Delhi, Public Health Foundation of India, Gurgaon, Haryana, India; 2 Department of Medical Statistics, London School of Hygiene and Tropical Medicine, London, UK; 3 Department of Nutrition, Harvard School of Public Health, Boston, Massachusetts, USA

**Keywords:** Childhood, Immunization, Urban poor

## Abstract

**Objectives:**

Aggregate data on childhood immunisation from urban settings may not reflect the coverage among the urban poor. This study provides information on complete childhood immunisation coverage among the urban poor, and explores its household and neighbourhood-level determinants.

**Setting:**

Urban poor community in the Southeast district of Delhi, India.

**Participants:**

We randomly sampled 1849 children aged 1–3.5 years from 13 451 households in 39 clusters (cluster defined as area covered by a community health worker) in 2 large urban poor settlements. Of these, 1343 completed the survey. We collected information regarding childhood immunisation (BCG, oral polio vaccine, diphtheria–pertussis–tetanus vaccine, hepatitis B and measles) from vaccination cards or mothers’ recall. We used random intercept logistic regression to explore the sociodemographic determinants of complete immunisation.

**Results:**

Complete immunisation coverage was 46.7% and 7.5% were not immunised. The odds of complete vaccination (OR, 95% CI) were lower in female children (0.70 (0.55 to 0.89)) and Muslim households (0.65 (0.45 to 0.94)). The odds of complete vaccination were higher if the mother was literate (1.6 (1.15 to 2.16)), if the child was born within the city (2.7 (1.97 to 3.65)), in a health facility ( 1.5 (1.19 to 2.02)), belonged to the highest wealth quintile (compared with the poorest; 2.46 (1.5 to 4.02)) or possessed a birth certificate (1.40 (1.03 to 1.91)). Cluster effect due to unmeasured neighbourhood factors expressed as median OR was 1.32.

**Conclusions:**

Immunisation coverage in this urban poor area was much lower than that of regional surveys reporting overall urban data. Socioeconomic status of the household, female illiteracy, health awareness and gender inequality were important determinants of coverage in this population. Hence, in addition to enhancing the infrastructure for providing mother and child services, efforts are also needed to address these issues in order to improve immunisation coverage in deprived urban communities.

**Trial registration number:**

CTRI/2011/091/000095.

**Strengths and limitations of this study**

- We report current estimates of childhood complete immunisation including hepatitis B vaccine coverage from representative urban poor communities in the Southeast of Delhi.
- The sample size was large and therefore our effect estimates for coverage and determinants were precise.
- We quantify unknown neighbourhood effects on this outcome using median ORs which are more intuitively understood.
- Based on the data, representative of only one district of Delhi.
- We did not capture appropriateness of timing of vaccination.

## Background

The WHO Expanded Programme on Immunization (EPI) recommends that all children receive one dose of BCG, three doses of diphtheria–pertussis–tetanus vaccine (DPT), three doses of oral polio vaccine (OPV), three doses of hepatitis B vaccine and one dose of measles vaccine.^1^ The coverage for these major vaccine-preventable diseases has risen significantly since EPI began in 1974 when the global vaccination coverage was only 5%. Despite this progress, an estimated 1.5 million children worldwide die each year of diseases that can be readily be prevented by these vaccines.^1^ The current goal as per the Global Vaccine Action Plan is to reach at least 90% of the population nationally, and at least 80% in every district.^2^

Receiving three doses of DPT is considered one of the key indicators of childhood vaccine coverage. By this metric, in 2013, India accounted for the single largest number of partially vaccinated children in the world. Of the 21.8 million children worldwide who did not receive three doses of DPT, 6.9 million were from India.^3^ According to the District Level Household and Facility Survey (2008; DLHS-3), 53.5% of children aged between 12 and 23 months in India were fully immunised for the six vaccine preventable diseases (hepatitis B not included), while 4.6% of children were not immunised at all.^4^ Among children living in urban areas, complete vaccination coverage was 63.1%.^4^ The coverage estimate from the Rapid Survey on Children (RSOC) undertaken by the Ministry of Women and Child Development and Unicef between November 2013 and May 2014 shows some encouraging trends with 65.3% of children (12–23 months) fully immunised for the country as a whole and 72% coverage among those living in urban areas.^5^ However, there is wide variation in this percentage both between and within Indian states. Coverage is also affected by several individual demographic characteristics such as literacy, gender of the child and socioeconomic position (SEP).^6^ Several demand side (socioeconomic, lack of awareness and cultural beliefs and distance to health facility) as well as supply side factors (poor quality of services, inadequate staffing and irregular supply of vaccines) have been suggested as potential reasons for the low immunisation coverage in India.^6^ ^7^

As per DLHS-3, in India's national capital Delhi, 67.3% of children aged between 12 and 23 months were completely immunised and 2.1% were not immunised at all.^8^ Delhi stood 15th among the 34 states and union territories of India with the best performing state having a coverage of 89.8% and the worst 13.3%.^4^ Another survey undertaken by Unicef in 2009 showed slightly different coverage rates in Delhi (complete immunisation (71.5%) and not immunised (7.3%)).^9^ Similar estimates have been shown from the RSOC (2013–2014) Delhi data (69.7% fully immunised and 4.8% not immunised) indicating a stagnation in complete immunisation coverage in the national capital since 2009.^10^

Moreover, the urban data from India are usually aggregates of urban slum and non-slum areas that mask socioeconomic inequalities. Within Delhi (National Family Health Survey (NFHS), 2008), there was substantial difference between complete immunisation percentages between slum (51.7%) and non-slum dwellers (67%).^11^ Further, the coverage estimates among the urban poor in these surveys is typically based on a very small sample. For example, in the NFHS-3, the total sample contributed by the urban poor of Delhi was just 46 and most other national surveys do not provide urban poor estimates. From the published literature, the following gaps in information regarding immunisation coverage in the urban poor population have been identified. The majority of recent studies from India conducted in urban poor settings (table 1), while providing estimates of immunisation coverage, do not include hepatitis B vaccine coverage in their definitions (with the exception of the two surveys from Delhi).^12^ ^13^ Further, most of these studies were small (median sample size of 380 participants) and did not look at determinants of complete immunisation using multivariable models. Also, the studies that used cluster sampling methods did not explore the extent of clustering of this outcome. Given that the determinants of complete immunisation are likely to differ between the urban poor and non-poor, larger samples are needed to explore context-specific factors affecting immunisation coverage among the poor.

**Table 1:** Prevalence estimates of complete immunisation in urban poor settlements from other surveys from India, Pakistan and Bangladesh

Place, state/year of survey*	Setting, sampling and sample size	Complete (C), partial (P), no (N) immunisation† (%)	Factors associated with no/partial immunisation
Bareilly, Uttar Pradesh^22^ (2010)	Urban slum 30×7 cluster sample (n=210)	C=61.9%, P=31.43%, N=6.67% BCG to measles attrition=32.8%	Unadjusted analysis: religion, education of mother and father
Lucknow, Uttar Pradesh^32^ (2005)	Urban slum WHO 30 sample method (n=510)	C=44.1%, P=32%, N=23.9% Overall dropout rate: 33.24%	Adjusted analysis: socioeconomic status, religion, birth order, place of childbirth, type of family
Lucknow, Uttar Pradesh^27^ (2012)	Attendees of Urban Health Centre (n=198)	C=74.7%, P=11.1%, N=14.1%	Unadjusted: larger households, place of childbirth, mother education
Lucknow, Uttar Pradesh^33^ (2013)*	Eight clusters (*Mohalla*) in city Random sample (n=450)	C=62.7%, P=24.4%, N=12.9% BCG to measles attrition=29%	Not explored
Rewa, Madhya Pradesh^34^ (2012–13)	Urban slum 30×7 cluster sample (n=210)	C=72.4%, P=21.9%, N=5.7%	Unadjusted: no association seen
Jamnagar, Gujarat^35^ (2005)	Urban slums 30×7 cluster sample (n=210)	C=73.3%, P=23.81%, N=2.86%	Not explored
National Capital territory, Delhi^12^ (2010)*	Random sample from 30 migrant well-settled colonies (n=407)	C=80.8%, N=4.9% C=60.2% (including hepatitis B vaccine), hepatitis B=68.4%	Not explored
Rewa, Madhya Pradesh^36^ (2013*)	Urban slum 30×7 cluster sample (n=210)	C=60.7%, P=32.7%, N=6.6% BCG to measles attrition=19.5%	Not explored
Mumbai, Maharashtra^23^ (2008)	Urban Slums Lot quality technique (n=352)	C=88.07%, N=11.9%	Unadjusted: gender, religion, mother and father education, mother and father occupation, SES score, birth order, presence of immunisation card and place of birth
Ahmedabad, Gujarat^37^ (2006)	30 slum clusters (n=138)	C=70.3%, P=29.7%, N=0% BCG to measles attrition=13.9%	Not explored
Bijapur, Karnataka^38^ (2011)	All eligible children from purposively chosen 7 slum clusters (n=155)	C=34.84%, P=62.54%, N=2.58% Overall attrition=57.05%	Not explored
West Delhi^25^ (2013)	2-stage probability-proportional-to-size cluster sampling (9 clusters) (n=670)	DPT 3 dose=80.5%	Adjusted analysis: health literacy of mothers
East Delhi^13^ (2003–2004)	Systematic random sampling from 2 urbanised villages (n=693)	C=41% Hepatitis B=24.3%	Adjusted analysis: place of childbirth, immunisation card, mother education
Dhaka, Bangladesh^39^ (2006–2007)	2 purposively sampled urban slum (random selection of children) (n=529)	C=43%, P=33%, N=2% Invalid doses=22%	Not reported
Pakistan^40^ (2002)	All infants living in neglected colony in Multan city (n=993)	C=18%, P=50.8%, N=31.2%	Unadjusted analysis: mother's literacy, father's literacy, household income, working mothers
Dhaka, Bangladesh^41^ (1995)	Zone 3 of Dhaka city, 5940 households containing 160 geographical clusters	C=38%	Adjusted analysis: number of living children, mother's education and employment status, distance to nearest immunisation centre

*Wherever the time of survey is not known, we have given the time of publication.

†The definition of complete immunisation was (three doses of OPV, DPT, one dose of measles and BCG) and the age group was from 12 to 23 months.

DPT, diphtheria–pertussis–tetanus vaccine; OPV, oral polio vaccine; SES, socioeconomic status.

This survey of under-5 children residing in the urban poor settlements of Delhi was conducted as part of a larger implementation research project ANCHUL (*A*nte *N*atal and *C*hild *H*ealth care in *U*rban S*L*ums) assessing the effectiveness of a complex intervention targeted at community health workers under Delhi State Health Mission, in improving usage of maternal, neonatal and child health (MNCH) services in urban poor settlements of Delhi. In this report, we present contemporary estimates of immunisation coverage and also analyse individual, household and neighbourhood determinants of complete immunisation in this urban poor community of Delhi.

## Methods

### Setting

The study areas are two large, purposively chosen urban poor settlements in the Southeast district of Delhi. This district has three subdivisions and is a relatively new revenue district carved out of the South district of Delhi in 2012.^14^ Two large representative urban poor settlements, namely *Lalkuan* and *Sangam Vihar* (B and C) blocks from *Sarita Vihar* subdivision of Southeast district, were chosen as the study areas, in consultation with the Delhi Government. Each of the study areas is served by a Primary Urban Health Centre (PUHC) situated within the settlement, which is equivalent to a primary health centre in the hierarchy of the healthcare facility structure. Since the larger study assessed the effectiveness of community healthcare workers in optimising MNCH care usage, we demarcated the two settlements into 39 clusters of ∼400 households each, based on the coverage area of each community health worker.

### Sampling

Our study population is a random sample of children aged between 12 and 42 months. We listed a total of 16 221 households in the study area, of which 13 451 completed the household survey. Of the 13 451 households, 22.8% had at least one child aged between 1 and 3.5 years. We desired to have a sample of 1500 children to obtain information on immunisation. Taking non-responses into account, we randomly chose 60% of households stratified by cluster (n=1849). We performed the random sampling using the runiform function of Stata V.13, after sorting the full data by clusters. In households that had more than one eligible child, we chose the youngest child. Trained field interviewers collected data after obtaining written informed consent from the mother or caregiver.

### Data collection

We collected household and neighbourhood information during the baseline household survey, the methodology for which is described elsewhere.^15^ Information on the place of birth of child, education and occupation of parents, immunisation history and any illness in the past 1 month was collected. We obtained details of BCG, 0–3 doses of OPV, DPT, hepatitis B vaccine and measles primarily from the vaccination card. In the absence of the card, information was obtained from the mother. Interviewers additionally checked for the BCG scar. All data were collected using electronic data capture via smartphones.^15^ This survey was conducted between February 2014 and April 2014.

### Measurements

On the basis of the information on vaccination, we categorised the child as ‘immunised’ if one dose of BCG and measles and three doses of DPT, OPV and hepatitis B vaccine had all been administered. If a child who had received at least one vaccine (but not all) was categorised as ‘partially immunised’, while a child who did not receive any vaccine was considered ‘not immunised’.

*Independent variables*: Hazardous location of the communities (a cluster that is located next to a garbage dump or open sewage drains or large waterbody), type of housing, sanitation facilities, water supply, electricity and house ownership were collected from each cluster by observation and from individual households to compute the cluster vulnerability score (ranging from 0 to 10 with higher values indicating a higher level of vulnerability) as described by Osrin *et al*.^16^ The distance to the PUHC from an arbitrary centre of the cluster was calculated in kilometres. Household-level socioeconomic scores were computed using information of dwelling characteristics and household possessions by the principal component analysis method, the details of which are found in Devasenapathy *et al*.^15^ The quintiles of the score were used to classify the households into five categories (poorest to least poor) of SEPs. Other sociodemographic indicators at the household level were religion, caste (scheduled caste or scheduled tribe/other backward class/general), type of family (nuclear/extended), family size (≤5/>5), duration of stay in Delhi (≤10/>10 years) and possession of the national identity card (*Aadhar* card). We also collected variables related to the child, namely education of parents (literate/illiterate (not enrolled at school)), gender of the child, birth order of child (first child or not) and place of birth (Delhi/outside Delhi), place of childbirth (facility/home).

### Statistical analysis

We present the descriptive data at the cluster, household and child level with continuous variables presented using means and SD, categorical variables as frequencies and percentages. We computed the prevalence estimates of vaccination coverage of individual vaccines and overall childhood immunisation, along with 95% CI which took clustering into account. We used random intercept logistic regression for exploring sociodemographic determinants for complete immunisation. As a first step, univariable analyses were performed with each of the demographic indicators and outcome. Gender of the child, literacy of mother and religion were a priori confounders. The multivariable model included the a priori confounders, cluster-level variables and other variables that had a p value <0.1in the univariable analysis. We present unadjusted and adjusted OR and 95% CI of all the variables that were included in the multivariable analysis. We considered a p value <5% as statistically significant. Interaction between gender of the child and religion and gender and SEP was explored as previous literature has shown gender disparity in immunisation to vary across religion and SEP. For the purpose of comparability with previous literature, the gender prevalence ratio of complete immunisation (girl/boy) was calculated. intracluster correlation coefficient (ICC) is the proportion of total variance in the outcome that is attributable to the cluster-level variance. However, for binary outcomes, the individual-level variance is in probability scale and cluster-level variance is in logistic scale, making the interpretation of ICC less intuitive. Hence, we also report the median ORs (MORs) for the null and the final model to quantify the area-level variance in the same scale as the effect estimates (ie, OR) as suggested by Merlo *et al*^17^ 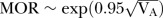; V_A_=cluster-level variance). MOR indicates the extent to which the individual probability of getting complete immunisation is determined by residential area. All analyses were done using STATA V.13 using the melogit command.

## Results

**Figure 1:**
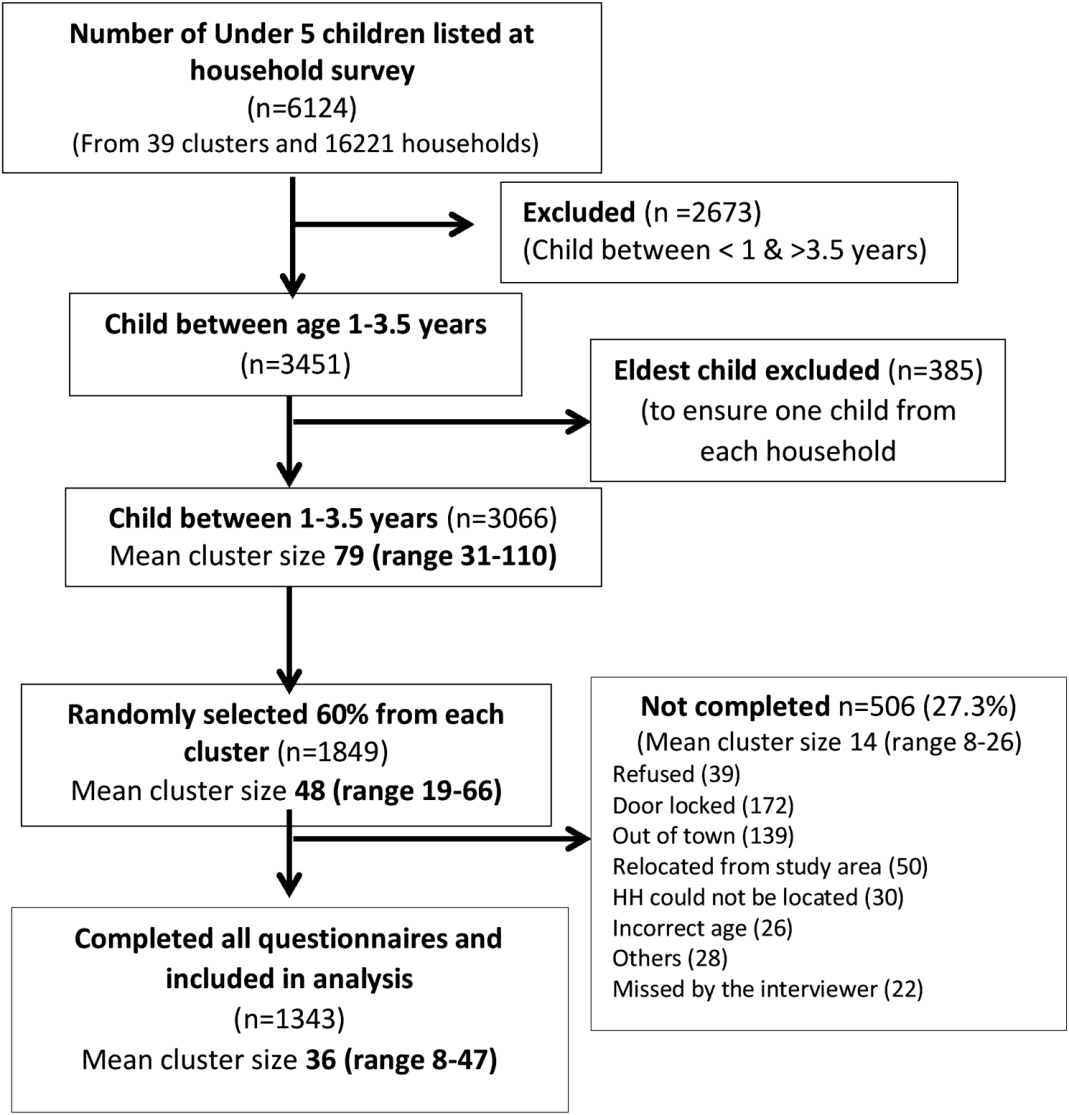
Sampling scheme for the immunisation survey. HH, households.

Of the 1849 randomly sampled households with one eligible child per household, the questionnaire was completed by 1343 mothers/caregivers. The reasons for non-response by 27.3% of the sample are listed in the flow chart (figure 1) with the most common reason being non-availability of a respondent. The household and sociodemographic characteristics of the study population are described in table 2. The study population was from a mature urban poor settlement with 90% living in Delhi for 10 years or more. Most parents availed treatment for their child from private clinics (80%) for common ailments. In our survey, we found 41 private clinics functioning in the study area. However, none of them reported providing childhood vaccination. Very few mothers were aware of government-run mother and child health schemes like Janani Suraksha Yojna (JSY; a cash transfer scheme for institutional delivery for below poverty line populations in Delhi) and Janani Shishu Suraksha Karyakram (JSSK; a scheme for providing free medical services to below poverty line mothers and neonates in Delhi); and only 3% of mothers mentioned the presence of a community health worker in their locality at the time of the survey. Non-responders in this survey differed from responders mainly in two important characteristics. They were likely to be poorer and were recent entrants to the locality (<5 years; see online supplementary appendix 1, table 1).

**Table 2:** Cluster-level, household-level and individual-level characteristics of the study sample

Cluster-level characteristics	N=39
Mean households per cluster (range)	36 (8–47)
Number of clusters with active NGO activity (%)	19 (49)
Mean cluster vulnerability score (SD)	2.9 (1.6)
Mean distance in km to the Primary Urban Health Centre (SD)	0.67 (0.32)
Total number of pharmacies	21
Clusters that have at least 1 pharmacy (%)	13 (33)
Total clinics in study area	41
Clusters that have at least 1 clinic (%)	30 (77)
Total number of *anganwadis* (government-run childcare centre) in 39 clusters	48
Clusters that have at least 1 anganwadi (%)	35 (90)
Clusters that have at least 1 school (%)	26 (67)
**Household-level characteristics**	**N=1343**
Muslim households (%)	205 (15.26)
Caste (%)	
Scheduled class/scheduled tribe	543 (40.43)
Other backward class	301 (22.41)
General	499 (37.16)
Nuclear family (%)	960 (71.48)
Mean family size (SD)	5.4 (2.2)
Households with family size >5 (%)	505 (37.6)
Possessing BPL card (%)	13 (0.97)
Possessing Aadhar card (national identity card) (%)	1030 (76.69)
Functional piped water facility within house (%)	1101 (81.98)
Underground drainage (%)	260 (19.36)
Living in Delhi for more than 10 years (%)	1187 (88.38)
Living in same locality for more than 5 years (%)	1115 (83.15)
Living in same house for equal/more than 3 years (%)	971 (72.30)
Socioeconomic quintiles (%)	
0 (poorest)	243 (18.09)
1	287 (21.37)
2	266 (19.81)
3	259 (19.29)
4 (least poor)	288 (21.44)
**Participant-level characteristics**	**N=1343**
Male child (%)	697 (51.90)
Mean birth order (SD)	2.24 (1.23)
Per cent of first child	423 (31.5)
Mean age of child in years (SD)	2.38 (0.73)
Day care (%)	
Home	1096 (81.61)
Anganwadi	137 (10.20)
Preschool/creche	110 (8.19)
Child born in Delhi (%)	998 (74.31)
Born in a facility (%)	864 (64.33)
Full term (%)	1313 (97.77)
Birth weight known (%)	708 (52.72)
Mean Birth weight in grams (SD)	2689 (639)
Possession of birth certificate (%)	1050 (78.18)
Mother	
Mean age in years (SD)	26.42 (4.01)
Literate (%)	1000 (74.46)
Employed (%)	65 (4.84)
Father	
Literate (%)	1208 (89.95)
Employed (%)	1308 (97.39)
Type of facility visited in the event of child illness (%)	
Private clinic	1084 (80.71)
Private hospital	106 (7.89)
Public health post	96 (7.15)
Government general hospital	57 (4.24)

BPL, below poverty line; NGO, non-governmental organisation.

10.1136/bmjopen-2016-013015.supp1 Supplementary appendix

### Vaccine coverage

**Figure 2:**
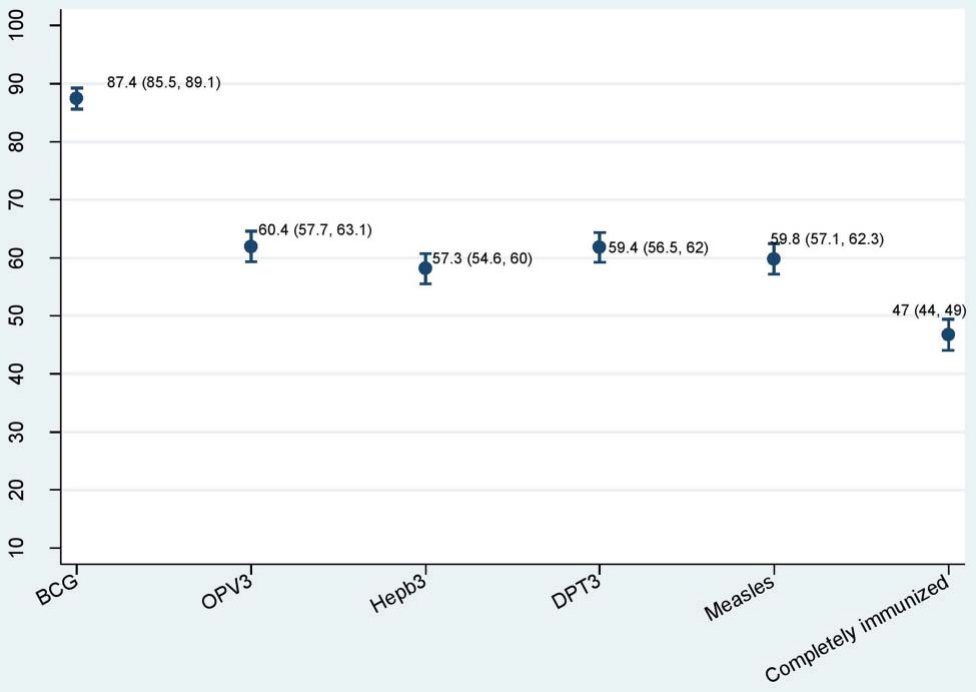
Immunisation coverage of five vaccines and complete immunisation (%, 95% CI). DPT, diphtheria–pertussis–tetanus vaccine; Hepb, hepatitis b; OPV, oral polio vaccine.

Sixty-four per cent of the mothers interviewed possessed an immunisation card. Of the 1343 children, 46.7% (95% CI 44% to 49.4%) were completely immunised for all doses of five vaccines including hepatitis B, 45.9% (95% CI 43.2% to 48.6%) were partially immunised and 7.5% (95% CI 6.2% to 9%) had not received any vaccination at all. Of the five vaccines, the coverage was highest (87.4%) for BCG and lowest for hepatitis B (57.3%). Three doses of DPT were completed by 59.4% of children (figure 2). The attrition rate of DPT was 4.6% from the first to second dose, 6.3% from the second to third dose and 9.9% from the first to third dose. The attrition rates were similar for OPV (4.6%, 5.5% and 9.1%) and were marginally higher for hepatitis B (5.9%, 6.8% and 12.2%).The overall attrition rate from BCG to measles was 36%. Half of the children (51%) had not received even one dose of vitamin A.

### Determinants of complete immunisation and cluster-level effects

**Figure 3:**
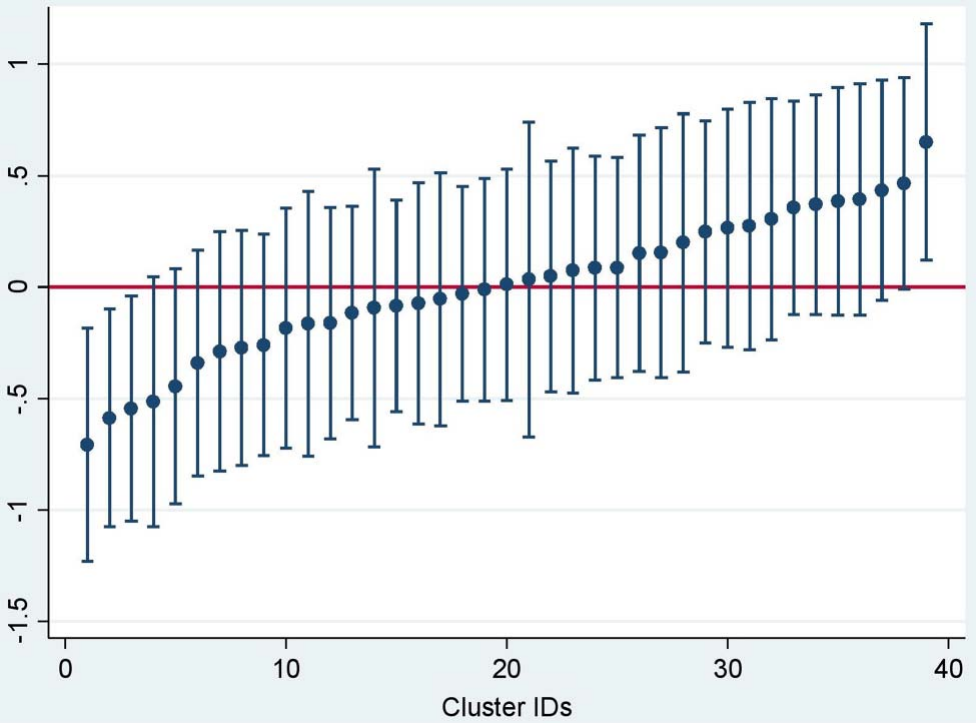
Variation in immunisation coverage by cluster plotted using cluster-level residuals.

Table 3 presents the crude and adjusted ORs defining the association between various sociodemographic indicators and complete immunisation. The odds of being completely vaccinated were lower for female children, children born to illiterate mothers, children in Muslim households, children in households belonging to lower SEP and children born outside Delhi. Children born in hospitals had higher odds of being vaccinated completely. Further, parents who were in possession of a birth certificate for their child were also the ones who were more likely to have their child completely immunised. The girl-to-boy complete immunisation coverage ratio was 0.78. We did not find any interaction between gender of the child and religion and gender of the child and SEP. The ICC for complete immunisation was 0.051 (95% CI 0.024 to 0.102), corresponding to an MOR of 1.49. Figure 3, a plot of the cluster-level residuals and their 95% CI against the study clusters, illustrates the variation in the immunisation coverage by cluster, again emphasising neighbourhood effects. The MOR of the full model with two cluster-level factors (vulnerability and distance to PUHC) was 1.32. This means that the odds of complete immunisation of a child randomly picked up from one cluster is 1.32 times higher when compared with a child randomly picked up from another cluster.

**Table 3:** Determinants of complete immunisation

Characteristics	Not/partially immunised n=716	Completely immunised n=627	Unadjusted OR (95% CI) p Value	Adjusted OR (95% CI) p Value
Gender				
Male	344 (48.04)	353 (56.30)	1	1
Female	372 (51.96)	274 (43.70)	0.70 (0.56 to 0.87) 0.002	0.70 (0.55 to 0.89) 0.003
Mother's age (years)				
≤25	359 (50.07)	340 (54.14)	1	1
>25	358 (49.93)	288 (45.86)	0.85 (0.68 to 1.06) 0.151	0.84 (0.64 to 1.09) 0.2
Birth order				
First child	198 (27.65)	225 (35.89)	1	1
Second or higher	518 (72.35)	402 (64.11)	0.69 (0.54 to 0.87) 0.002	0.83 (0.62 to 1.11) 0.21
Mother's literacy				
Illiterate	239 (33.38)	104 (16.59)	1	1
Literate	477 (66.62)	523 (83.41)	2.44 (1.86 to 3.20) <0.001	1.58 (1.15 to 2.16) 0.004
Father's literacy				
Illiterate	91 (12.71)	44 (7.02)	1	1
Literate	625 (87.29)	583 (92.98)	1.74 (1.17 to 2.57) 0.005	0.90 (0.58 to 1.41) 0.66
Family type				
Non-nuclear	172 (24.02)	211 (33.65)	1	1
Nuclear	544 (75.98)	416 (66.35)	0.59 (0.46 to 0.76) <0.001	0.88 (0.63 to 1.22) 0.44
Family size				
≤5 members	463 (64.7)	375 (59.8)	1	1
>5 members	253 (35.3)	252 (40.2)	1.23 (0.98 to 1.54) 0.073	0.81 (0.60 to 1.09) 0.17
Place of birth				
Outside Delhi	254 (35.47)	91 (14.51)	1	1
Within Delhi	462 (64.53)	536 (85.49)	3.45 (2.60 to 4.56) < 0.001	2.7 (1.97 to 3.65) <0.001
Place of childbirth				
Home	310 (43.30)	169 (26.95)	1	1
Facility	406 (56.70)	458 (73.05)	1.95 (1.53 to 2.47) <0.001	1.55 (1.19 to 2.02) 0.001
Religion				
Non-Muslim	580 (81.01)	558 (89.00)	1	1
Muslim	136 (18.99)	69 (11.00)	0.54 (0.39 to 0.76) <0.001	0.65 (0.45 to 0.94) 0.023
Caste				
SC/ST (ref)	295 (41)	248 (40)	1	1
OBC	176 (24.6)	125 (20)	0.83 (0.61 to 1.11)	0.89 (0.64 to 1.25)
General	245 (34.2)	254 (40.5)	1.16 (0.90 to 1.5) 0.075	1.09 (0.82 to 1.44) 0.5
Socioeconomic position				
0 (poorest)	177 (24.72)	66 (10.53)	1	1
1	180 (25.14)	107 (17.07)	1.56 (1.06 to 2.27)	1.3 (0.87 to 1.97)
2	142 (19.83)	124 (19.78)	2.34 (1.60 to 3.43)	1.57 (1.03 to 2.38)
3	118 (16.48)	141 (22.49)	3.23 (2.20 to 4.74)	1 (1.29 to 3.097)
4 (least poor)	99 (13.83)	189 (30.14)	4.85 (3.29 to 7.16) <0.001	2.46 (1.5 to 4.02) 0.005
Living in Delhi				
<10 years	105 (14.66)	51 (8.13)	1	1
More than10 years	611 (85.34)	576 (91.87)	2.01 (1.39 to 2.90) <0.001	1.1 (0.72 to 1.67) 0.66
Aadhar card				
No	199 (27.79)	114 (18.18)	1	1
Yes	517 (72.21)	513 (81.82)	1.90 (1.45 to 2.50) <0.001	1.09 (0.79 to 1.5) 0.59
Birth certificate				
No	201 (28)	92 (14.7)	1	1
Yes	516 (72)	536 (85.3)	2.28 (1.71 to 3.02) <0.001	1.40 (1.03 to 1.91) 0.033
Cluster vulnerability score (0–10)	−	−	0.85 (0.77 to 0.93) 0.001	0.91 (0.81 to 1) 0.07
Distance to PUHC in km	−	−	0.90 (0.53 to 1.5) 0.69	1.12 (0.67 to 1.88) 0.65

ICC for immunisation was 0.05 (95% CI 0.024 to 0.102); MOR=1.5. Conditional ICC of the final model=0.026, MOR=1.32.

(MOR is a measure of residual cluster-level heterogeneity. When we compare two children from randomly chosen different clusters, with the same covariates, MOR is the MOR between the child of higher odds and child of lower odds.)

ICC, intracluster correlation coefficient; MOR, median OR; OBC, other backward class; PUHC, Primary Urban Health Centre; SC, scheduled caste; ST, scheduled tribe.

In order to explore the possibility of bias due to recall by the mother regarding immunisation status, we performed another multivariable analysis (results not shown here) using only those respondents who had an immunisation card. We found that the results did not change except for a reduction in precision around the estimates.

## Discussion

Less than half of the children between 1 and 3.5 years of age were completely immunised with the five vaccines. Our estimates were less than the overall state-level average of 70% reported in the RSOC (2014)^10^ and also fall far short of the goal of Global Vaccine Action Plan of 90% coverage. Our estimates are contemporary and reliable for the given area as they were derived from an adequately sized random sample drawn from two large urban poor settlements encompassing a population of ∼80 000 using robust data collection methods. This study was an implementation research project which involved working closely with the state government, and we were unable to randomly sample clusters from all over Delhi state. While this may potentially affect the generalisability of our findings, we are confident that the study areas are representative of a typical urban poor settlement since the locations included in our study were recommended to us by the government as neglected and underserved populations.

### Coverage

The RSOC survey (2014) and Unicef survey (2009) report results for the urban population as a whole without further stratification of coverage among the urban poor and non-poor population. Therefore, the low coverage rates found in our study could be due to the hidden discrepancy that exists in the immunisation coverage between the urban poor and non-poor populations residing in a large metropolitan city of India. The Unicef survey (n=585) reported BCG, OPV, DPT, hepatitis B and measles coverage as 89.1%, 76%, 79%, 64.5% and 83.3%, respectively,^9^ and the RSOC reported only the DPT 3 (74.5%) and measles first dose (84.7%). In our study, other than BCG, none of the other vaccines had a coverage over 60%. However, the attrition rates of DPT 2–3 were marginally higher in the Unicef data and RSOC data (9% and 17.1%)^9^ ^10^ than in our study (6.3%).

The coverage was low in our study compared with most other developing country settings (table 1). Heterogeneity of these estimates across studies highlights the need for periodic local surveys for better implementation of immunisation programmes. All studies found ‘ignorance’ (regarding schedule and importance of vaccination) to be the most common reason, which led to misconceptions and an unwillingness to spend time and money for immunisation. Non-availability was reported by a very small percentage, indicating the predominance of demand side issues rather than supply side issues.

Hepatitis B vaccine was included in India's Universal Immunization Programme (UIP) in 2001 and piloted in 33 districts and 14 cities. It was subsequently rolled out in 10 states by 2008–2009 and all over the country by 2011. In a survey from East Delhi in 1999,^18^ three doses of hepatitis B were completed by 14% of children, which improved to 24.3% in another survey in 2003^13^ in the same district. This and other data had led to apprehensions regarding the sustainability of hepatitis B vaccination in India's UIP.^19^ However, in our study, the coverage of this vaccine was close to the well-established vaccines OPV and DPT (60%), emphasising the success of implementation of hepatitis B vaccine over time.

### Determinants

Most previous surveys in urban poor settlements were not large enough to perform an adjusted analysis for exploring the determinants. In our study, gender of the child, religion, mother's literacy and SEP of the household were strong sociodemographic indicators for complete immunisations.

*Gender inequity*: The girl-to-boy complete immunisation coverage ratio was 0.78 in our study population and did not vary across religions and SEP of household. Girl-to-boy coverage ratio of <1 was found across most states in India and this trend has not changed over the years in spite of increasing vaccine coverage.^20^ ^21^ On the basis of the NFHS-3 data, Singh^7^ showed that gender inequity in vaccination was high in the Indian states of Punjab (0.83), Delhi (0.83), Haryana (0.90), Bihar (0.88) and Uttar Pradesh (0.92). Further, there was also a discouraging trend in gender equality over time in the northeast, west and southern regions of India, which had low gender inequality in 1992.^7^

This calls for context-specific approaches to address gender discrimination in immunisation programmes, especially in communities disfavouring girl children.

*Religion*: Completion of the childhood immunisation schedule was lower in Muslim households compared with non-Muslim households and this finding is concordant with other previous surveys in India.^22^ ^23^ Religious beliefs affecting immunisation coverage is seen in low-income and middle-income countries as well as in high-income countries.^24^

*SEP of the household*: In an urban poor setting, our data showed a clear trend across the SEP gradient with the odds of immunisation to be 2.5 times higher among the less poor compared with the poorest. All nationally representative surveys have shown an urban poor/non-poor gradient but have not explored the gradient among the urban poor. Johri *et al*^25^ did explore association of SEP of households and completion of three doses of DPT among the urban poor, but did not find a statistically significant association like in our study. We have previously shown such a gradient in the same community for reproductive healthcare services.^15^ This emphasises the varying degrees of vulnerability even among the poorest of the poor communities and indicates the need to identify them for targeted interventions.

*Literacy and awareness*: Mother's literacy was strongly associated with immunisation (unlike father's literacy) in our adjusted model. The association of mother's literacy with immunisation has been previously demonstrated by Johri *et al*.^25^ Using nationally representative data, Vikram *et al*^26^ showed human capital (health knowledge) among mothers with primary education and cultural capital (communication skill) among mothers with secondary and college education as pathways that mediate relationship between education and child immunisation.

We did not measure health awareness in our survey. However, we considered the possession of a childbirth certificate to be a proxy measure for general awareness which was strongly associated with complete immunisation. The possession of a birth certificate can potentially be used as a simple tool to identify vulnerable households and future evaluations can include this in their survey.

*Place of childbirth*: This is concordant with the findings from other studies which showed a strong association of antenatal visits and hospital-based childbirths with future child immunisation practices.^22^ ^23^ ^27^ The initiative taken by the government of India of having the antenatal card combined with the child card capturing immunisation and growth milestones may encourage mothers to immunise their newborns appropriately.^28^

*Neighbourhood effects*: Since the MOR is more than 1 in our data, it means that the area of residence (cluster) would be relevant for understanding variations of the individual probability of complete immunisation. This is a measure of residual heterogeneity between clusters, indicating that there were several other unmeasured neighbourhood effects acting on this outcome. This finding and the socioeconomic gradient of household we demonstrate are important for policymakers as they highlight the heterogeneity within the poor. The cluster-level heterogeneity and its association with vaccine coverage have not been previously reported. This may be one factor underlying the frequently reported gap between programme coverage and impact, because it is the poor within the poor who may remain underserved. The study therefore underscores the need to address heterogeneity at the programmatic level and a ‘one-size-fits-all’ approach may be detrimental, especially in poor urban areas with rapidly changing populations. In spite of the potentially limited generalisability of our findings to all the urban poor settlements of Delhi, the public health message of our paper is an important one, especially in the era of the Sustainable Development Goals where equity and integrated and targeted programmes are strongly encouraged.

Other limitations of our study are that we did not capture appropriateness of timing of vaccinations and information on supply side issues that could have existed during the survey period. Further, around 36% of the information regarding immunisation was obtained from mother's recall, the correctness of which cannot be verified.

To sum up, our study provides insights into the rates and determinants of immunisation uptake by urban poor communities. We also report estimates of hepatitis B vaccine coverage which have so far been only infrequently reported. Further, our study demonstrates considerable cluster-level variation in immunisation coverage attributable to certain measured and unmeasured cluster-level factors, which will require further exploration and research.

### Strategies to improve coverage

**Figure 4:**
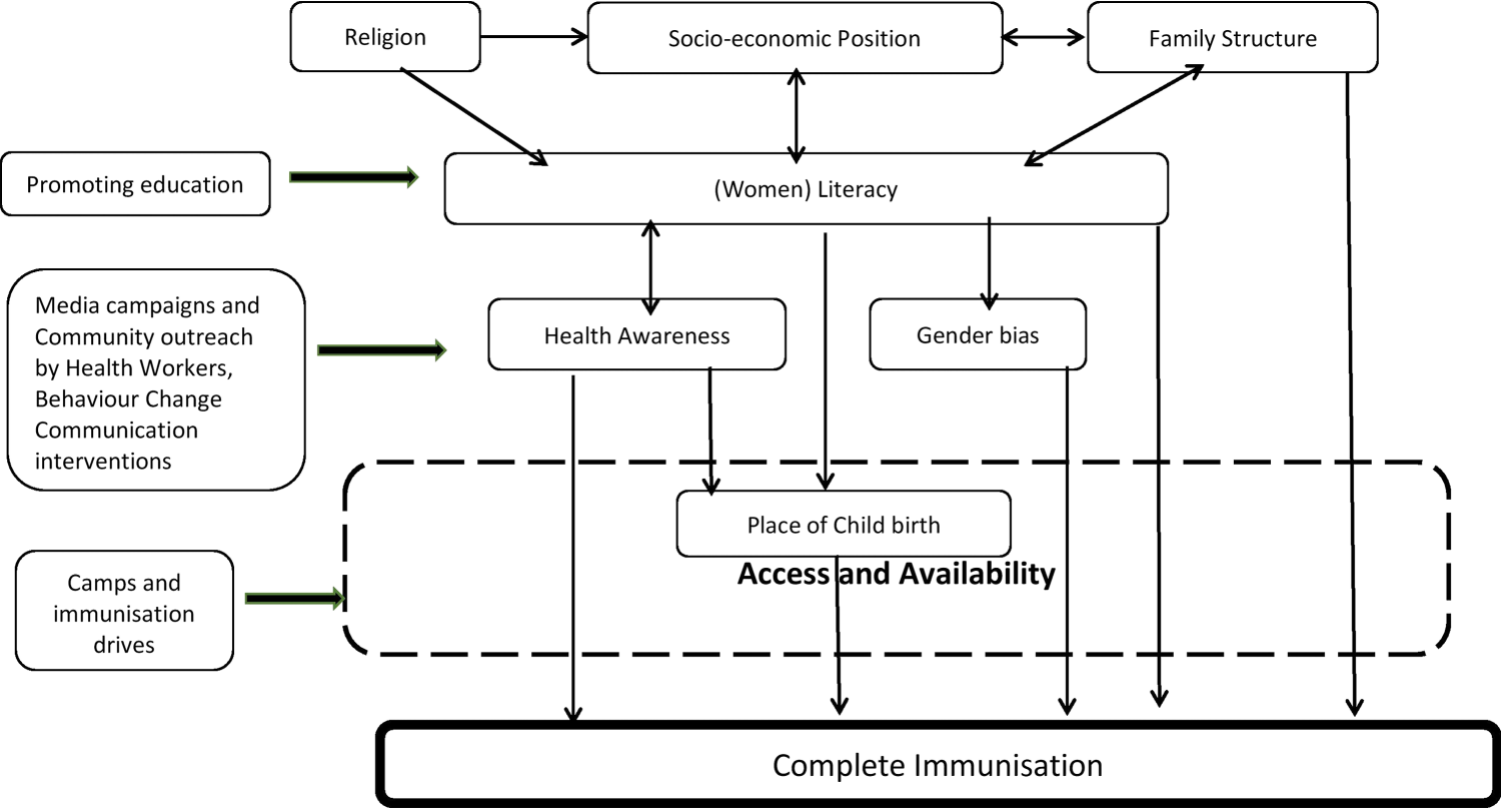
Determinants and mechanism of action of interventions.

India has made remarkable progress in becoming self-sufficient in vaccine manufacturing and availability, resulting in significant improvement in immunisation coverage through the implementation of the UIP.^29^ However, the data from waves of NFHS, DLHS and the latest RSOC indicate a stagnation in coverage rates after the late 1990s and the continuing existence of socioeconomic inequities in coverage.^6^ The Ministry of Health and Family Welfare of India launched Mission Indradhanush on 25 December 2014 with the aim of expanding immunisation coverage to all children across India by the year 2020.^30^ This mission includes four vaccine campaigns in a year with a special focus on poor performing districts. Further, in Delhi, urban ASHAs (Accredited Social Health Activist), akin to rural ASHAs, have been gradually introduced in the urban poor communities since 2007–2008 to improve uptake of services by the community. However, there have been conflicting results regarding the cost-effectiveness of involving community health workers in improving immunisation coverage.^31^ While campaigns and community outreach programmes have a role in increasing immunisation uptake and coverage, efforts to improve female literacy may be more sustainable and effective. Higher literacy among women may result in improved decision-making capacity and ability to overcome social and cultural barriers (illustrated in figure 4). Initiatives to improve education of the girl child are, in the long term, likely to show a wider and sustainable impact on all dimensions of health of the family and society.

## Conclusion

Our study findings confirm the poor immunisation coverage among the urban poor population and existence of important modifiable factors influencing vaccine uptake. Identifying and targeting vulnerable clusters and households within urban settlements via community-based outreach programmes are very much required as an interim effort and are vital to improving stagnant coverage rates. Since lack of awareness and social–cultural beliefs play a major role in the decision-making of families in vaccinating their children, overcoming these social barriers by improving female literacy and addressing lack of awareness or motivation, through professionally designed behaviour change communication interventions, will go a long way in improving child health in India.
