# Supplementary material for: Determinants of childhood immunisation coverage in urban poor settlements of Delhi, India: a cross-sectional study
Source: BMJ Open. 2016 Aug 26;6(8):e013015. doi: 10.1136/bmjopen-2016-013015 (PMC5013380; doi:10.1136/bmjopen-2016-013015)
Supplement: Supplementary appendix [file bmjopen-2016-013015supp_appendix.pdf]

Appendix 1: Table 1: Baseline household demographic characteristics comparison between those who completed and not completed the baseline survey

**Household demographic characteristics**	Completed U5 baseline survey n= 1343	Not completed U5 baseline survey n= 506*	P value
Muslim households (%)	205 (15.26)	88 (17.5)	0.197
Caste (%)			
Scheduled Caste/Scheduled Tribe	543 (40.43)	173 (34.39)	0.002
Other Backward Class	301 (22.41)	97 (19.28)	
General	499 (37.16)	233 (46.32)	
Nuclear family (%)	960 (71.48)	378 (75.15)	0.376
Mean family size (SD)	5.4 (2.2)	5.3 (2.6)	0.49
Possessing BPL card (%)	13 (0.97)	5 (0.99)	0.960
Possessing Aadhar card (%)	1030 (76.69)	337 (67.00)	< 0.001
Functional piped water facility (%)	1101 (81.98)	404 (80.32)	0.412
Underground drainage type (%)	260 (19.36)	98 (19.48)	0.177
Living in Delhi for more than 10 year (%)	1187 (88.38)	429 (85.29)	0.073
Living in same locality for more than 5 year (%)	1115 (83.15)	382 (76.10)	0.001
Socio-economic category			
0 (Poorest)	243 (18.09)	119 (23.66)	0.001
1	287 (21.37)	129 (25.65)	
2	266 (19.81)	101 (20.08)	
3	259 (19.29)	73 (14.51)	
4 (Least poorest)	288 (21.44)	81 (16.10)	

***Household demographic data missing for three households**
